# Oxaliplatin reacts with DMSO only in the presence of water†

**DOI:** 10.1039/c7dt01628j

**Published:** 2017-06-27

**Authors:** Hristo P. Varbanov, Daniel Ortiz, Doris Höfer, Laure Menin, Mathea S. Galanski, Bernhard K. Keppler, Paul J. Dyson

**Affiliations:** Institut des Sciences et Ingénierie Chimiques, Ecole Polytechnique Fédérale de Lausanne (EPFL) Lausanne Switzerland paul.dyson@epfl.ch; Institute of Inorganic Chemistry, University of Vienna Waehringer Strasse 42 A-1090 Vienna Austria hristo.varbanov@univie.ac.at

## Abstract

Herein we show that oxaliplatin reacts rapidly with DMSO in aqueous solutions, despite being stable in pure DMSO and pure water. Furthermore, the reactivity of the clinically applied Pt(ii) drugs in water/DMSO and PBS/DMSO mixtures, and the nature of the species formed were investigated by MS, NMR and RP-HPLC techniques.

## Abstract 2

Oxaliplatin is stable in pure water and pure DMSO, but reacts rapidly in a mixture of the two solvents.

## Graphical abstract

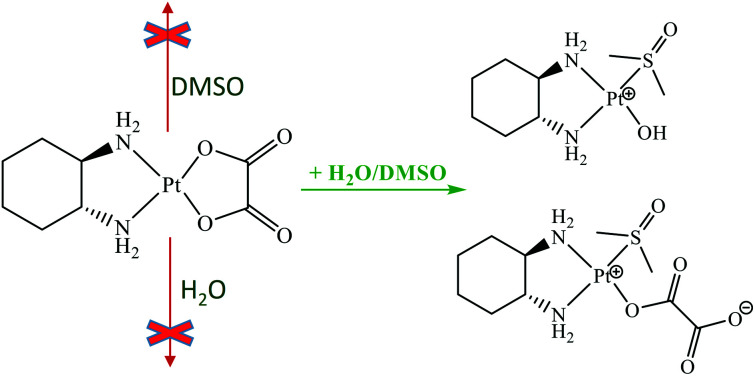

Cisplatin ([Pt(NH_3_)_2_Cl_2_]), carboplatin ([Pt(NH_3_)_2_(CBDCA)], where CBDCA = 1,1-cyclobutanedicarboxylate), and oxaliplatin ([Pt(DACH)(Ox)], where DACH = (1*R*,2*R*)-1,2-diaminocyclohexane and Ox = oxalate), are amongst the most effective and widely used anticancer drugs.^1,2^ They continue to be investigated in numerous preclinical and clinical studies in different combinations with other non-platinum drugs,^3–5^ and are widely used as benchmarks against which new putative metal-based compounds are compared.

These above-mentioned studies are usually conducted in aqueous media, often in the presence of dimethylsulfoxide (DMSO), which is probably the most commonly employed organic solvent in drug research and biological chemistry, due to its ability to dissolve/solubilize a large variety of therapeutically active molecules, its low toxicity and good miscibility with water.^6–8^ DMSO is also frequently used in experimental (laboratory and drug screening) settings involving Pt drugs,^9^ despite its known reactivity with cisplatin and subsequent effects on cell viability.^10–13^ Hall *et al.* reiterated this problem in a recent Cancer Research article entitled, “Say No to DMSO: Dimethylsulfoxide Inactivates Cisplatin, Carboplatin, and Other Platinum Complexes”.^9^ Therein, the authors examined the effects of DMSO on the *in vitro* biological activity of the clinically approved Pt-drugs, as well as of some relevant experimental complexes. A significant decrease in the cytotoxicity of cisplatin and related dichloridoplatinum(ii) complexes in several cancer cell lines was observed when their stock solutions were prepared in DMSO compared to the respective clinical formulations. Such findings are corroborated by the known lability of monodentate leaving groups such as chloride and the affinity of Pt(ii) to nucleophilic sulphur donors.^14–16^ Dicarboxylatoplatinum(ii) complexes such as carboplatin and oxaliplatin possess lower reactivity, respectively rate of exchange of the bidentate ligand compared to cisplatin^17–19^ and therefore a smaller effect of DMSO on their anticancer activity could be expected. Accordingly, Yi and Bae showed that cisplatin completely loses its activity against A2780 cells after 8 days of storage in DMSO (at 4 °C), while the potency of carboplatin and oxaliplatin is only slightly affected under the same conditions.^20^ Nevertheless, Hall *et al.* reported a 3- to 10-fold decrease of the cytotoxic activity of carboplatin when dissolved in DMSO whereas, intriguingly, no effect or only a slight potentiating effect of DMSO on the cytotoxicity of oxaliplatin was found. Furthermore, the authors confirmed (by means of ESI MS) the formation of Pt-DMSO species in DMSO solutions of cisplatin and carboplatin, but not for oxaliplatin.^9^

Using ESI MS, we noticed that oxaliplatin is stable in pure DMSO, but reacts rapidly with DMSO in aqueous solutions, prompting us to further investigate the reactivity of oxaliplatin (in comparison with cisplatin and carboplatin) in DMSO/water mixtures. The stability of cisplatin, carboplatin and oxaliplatin in DMSO-*d*_6_ was monitored over 48 h at RT using ^1^H NMR spectroscopy. As expected, cisplatin undergoes fast solvolysis (Fig. S1_a,d_†), whereas only minor changes were observed for carboplatin and oxaliplatin over 48 h of incubation (Fig. S1_b,c_†). These differences, *i.e.* a fast reaction between cisplatin and DMSO (Fig. S2†) and the high stability of carboplatin and oxaliplatin under the same conditions, were confirmed also by ESI MS. After 30 h in DMSO, only trace amounts (relative intensity of <1%) corresponding to Pt-DMSO adducts (*i.e.* [Pt(NH_3_)(DMSO)(CBDCA) + Na]^+^ and [Pt(NH_3_)_2_(CBDCA)(DMSO) + Na]^+^ for carboplatin; [Pt(DACH)(Ox)(DMSO) + H]^+^ for oxaliplatin) were identified. In contrast, MS signals characteristic for oxaliplatin rapidly diminish in aqueous solutions containing 1–10% DMSO with the growth of new peaks corresponding to DACH-Pt species, featuring one DMSO and one monodentate oxalate or hydroxide ligand (see Table 1, Scheme 1 and Fig. S3†).

**Scheme 1:**
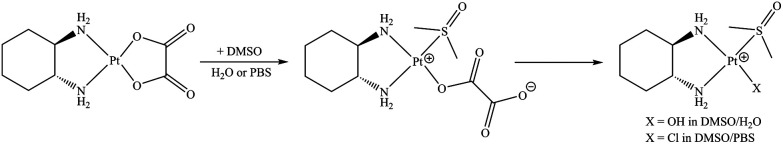
Formation of Pt-DMSO adducts in DMSO/H_2_O and DMSO/PBS (pH = 7.4) solutions of oxaliplatin.

**Table 1:** Pt-DMSO adducts identified in the ESI+ mass spectra of carboplatin and oxaliplatin after 24 h of incubation in DMSO/water (1 : 10)

Molecular species	Formula	Observed mass^a^	Theoretical mass	Error (ppm)
**Carboplatin**				
[Pt(NH_3_)_2_(CBDCA)(DMSO) + H]^+^	C_8_H_19_N_2_O_5_PtS	450.0664	450.0657	1.5
[Pt(NH_3_)_2_(CBDCA)(DMSO) + Na]^+^	C_8_H_18_N_2_O_5_PtSNa	472.0483	472.0476	1.5
[Pt(NH_3_)(DMSO)(CBDCA) + H]^+^	C_8_H_16_NO_5_PtS	433.0393	433.0391	0.5
[Pt(NH_3_)(DMSO)(CBDCA) + Na]^+^	C_8_H_15_NO_5_PtSNa	455.0218	455.0211	1.1
[Pt_2_(NH_3_)_3_(DMSO)(CBDCA)_2_ + H]^+^	C_14_H_28_N_3_O_9_Pt_2_S	804.0851	804.0836	1.8
[Pt_2_(NH_3_)_3_(DMSO)(CBDCA)_2_ + Na]^+^	C_14_H_27_N_3_O_9_Pt_2_SNa	826.0672	826.0656	1.9

**Oxaliplatin**				
[Pt(DACH)(OH)(DMSO)]^+^	C_8_H_21_N_2_O_2_PtS	404.0972	404.0966	1.4
[Pt(DACH)(Ox)(DMSO) + H]^+^	C_10_H_21_N_2_O_5_PtS	476.0817	476.0813	0.9
[Pt(DACH)(Ox)(DMSO) + Na]^+^	C_10_H_20_N_2_O_5_PtSNa	498.0635	498.0633	0.6
[Pt(DACH)(Ox)(DMSO) + K]^+^	C_10_H_20_N_2_O_5_PtSK	514.0373	514.0372	0.5

a Observed/theoretical masses are given for the monoisotopic peaks.

The reaction between oxaliplatin, DMSO and water was further studied by dissolving oxaliplatin (1 mM) in anhydrous DMSO and diluting the solution with water (1 : 20). The dilution was rapidly injected into the MS (for further details check the Experimental section in the ESI†). The evolution of the peaks corresponding to Pt-DMSO adducts formed (Table 1) *versus* the peaks corresponding to intact oxaliplatin was recorded in real time over 30 min. As shown in Fig. 1, the relative intensity of the signals corresponding to native oxaliplatin ([Pt(DACH)(Ox) + (H/Na/K)]^+^ at *m*/*z* 398.0674 (+H^+^), 420.0493 (+Na^+^) and 436.0233 (+K^+^)) drops rapidly to 33.5% in 29 minutes whereas the intensity of the peak attributed to [Pt(DACH)(OH)(DMSO)]^+^ increases to 63.2% in the same period. The different species of the type [Pt(DACH)(Ox)(DMSO) + (H/Na/K)]^+^ are formed faster (7.2% at 0.8 minutes), but remain below 9% during the entire experiment.

**Fig. 1:**
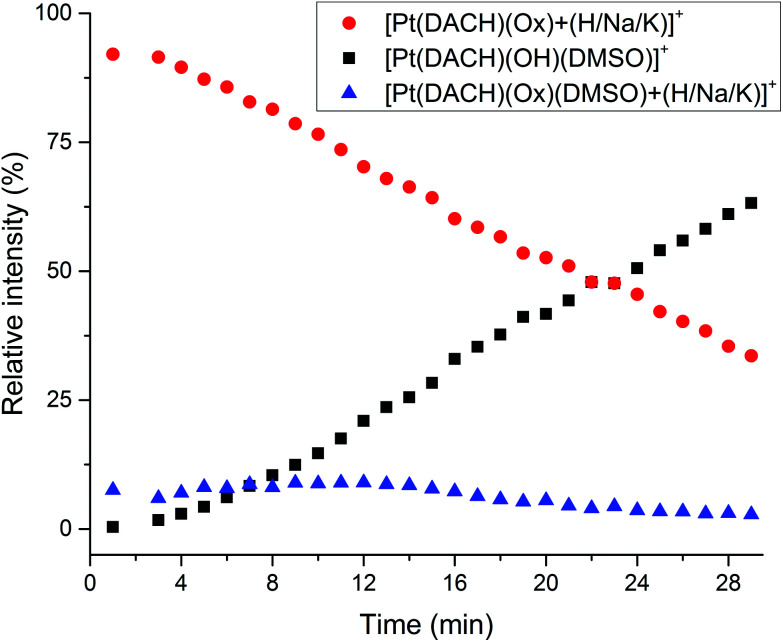
Relative intensity of the peaks corresponding to intact oxaliplatin ([Pt(DACH)(Ox) + (H/Na/K)]^+^, red 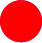), [Pt(DACH)(OH)(DMSO)]^+^ (black ■) and [Pt(DACH)(Ox)(DMSO) + (H/Na/K)]^+^ (blue 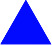) as a function of time in the ESI MS from a solution of oxaliplatin in DMSO after dilution with water (1 : 20). See Table 1 for details of peak assignments.

Next, we studied the respective clinical formulations of the Pt drugs (*i.e.* cisplatin – 3.3 mM in 0.9% saline; carboplatin – 27 mM in 5% glucose solution and oxaliplatin – 12.6 mM in 5% glucose solution) and diluted them with PBS (pH = 7.4)/DMSO or water/DMSO mixtures with different concentrations of DMSO (0–10%) to give a final concentration of the complexes of 1 mM. The formation of DMSO adducts at RT was followed by recording ESI MS (after the respective dilutions with the water/MeOH (10 : 1) mixture) at different time points for a period of 2 h (for cisplatin) and 40 h (for carboplatin and oxaliplatin). Cisplatin reacted with DMSO comparatively quickly in pure DMSO and in a mixture with water (Fig. S2†). In the case of carboplatin and oxaliplatin (both stable in pure DMSO and pure water) the rate of formation of DMSO adducts was dependent on the amount of DMSO in the solvent mixture, as shown in Fig. 2 for oxaliplatin. Oxaliplatin reacts almost completely to form mono-DMSO adducts within few hours in water/DMSO (10 : 1). DMSO coordinates to the Pt center, which causes the opening of the oxalato ring. The monodentately bound oxalate is further cleaved and exchanged with OH in H_2_O/DMSO or Cl in PBS/DMSO mixtures (Scheme 1, Fig. S3–S5†), and the reaction is considerably faster in PBS/DMSO (see Fig. 2, S4 and S6†). The rate enhancement in PBS/DMSO (compared to H_2_O/DMSO) may be caused by the coordination of Cl to the Pt center^21,22^ before or after DMSO coordination and/or the effect of phosphate and pH.

**Fig. 2:**
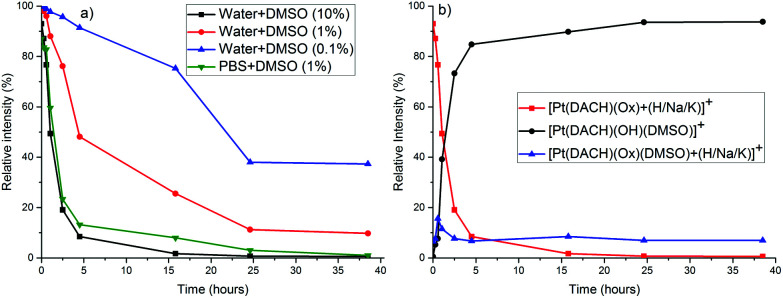
(a) Change in the relative intensity of the MS peaks corresponding to intact oxaliplatin as a function of time in a clinical formulation of oxaliplatin (12.6 mM in 5% glucose solution), diluted with different water/DMSO and PBS/DMSO mixtures as determined by ESI MS. (b) Time-dependent change in the relative intensity of peaks corresponding to the species formed in a clinical formulation of oxaliplatin after dilution with water/DMSO (10 : 1) as observed in the ESI MS+ spectra.

Carboplatin reacts with DMSO in water/DMSO mixtures, albeit at a much slower rate than oxaliplatin. After 48 h of incubation in water/DMSO (10 : 1), approximately 50% of carboplatin reacted to form adducts with DMSO, while in the (100 : 1) mixture the conversion was <20% (Fig. S7†). ESI MS spectra revealed the formation of several species, with the peak corresponding to [Pt(NH_3_)(DMSO)(CBDCA)] (also reported by Hall *et al.*^9^) being the most abundant. Peaks corresponding to adducts where DMSO causes the opening of the CBDCA ring (*e.g.*, [Pt(NH_3_)_2_(CBDCA)(DMSO) + H]^+^), as well as dimeric species, were also detected (see Tables 1 and S1 and Fig. S8†). The existence of at least three (incl. parent carboplatin) species in solution after 40 h of incubation was also confirmed by ^13^C and ^195^Pt NMR spectroscopy and RP-HPLC (Fig. S9†). All species detected during the time-dependent ESI MS experiments with cisplatin, carboplatin and oxaliplatin are listed in Table S1.†

To further verify and also gain deeper insight into the reaction between oxaliplatin and DMSO in DMSO/water mixtures, time-dependent ^13^C NMR spectroscopic studies using oxaliplatin with a ^13^C_2_-labeled oxalato ligand were performed. The reaction could be easily monitored following the changes in the 160–180 ppm region, where resonance(s) attributable to the oxalate carbon atoms are found. The signal characteristic for parent oxaliplatin decreases with time, and four signals belonging to monodentate ^13^C_2_-labeled oxalate (^1^*J*_C,C_ = 82.5 Hz) increase; a sixth signal corresponding to free oxalate was also observed in most cases (Fig. 3). The ^13^C NMR data corroborates with oxaliplatin-DMSO adducts observed in the ESI MS experiments. However, according to NMR spectra, the formation of the DMSO adduct with monodentate oxalate appears to dominate over the complete release of oxalate and the formation of [Pt(DACH)(OH)(DMSO)]^+^ (Fig. S10†). These observations were substantiated by RP-HPLC-MS experiments (Fig. S5†). The observed difference between ESI MS and NMR/LC-MS data may be attributed to the easier detection of the naturally charged [Pt(DACH)(OH)(DMSO)]^+^ complex compared to the neutral [Pt(DACH)(Ox)(DMSO)] species, reflected in the higher abundance in the positive ion ESI mass spectra of the former complex.

**Fig. 3:**
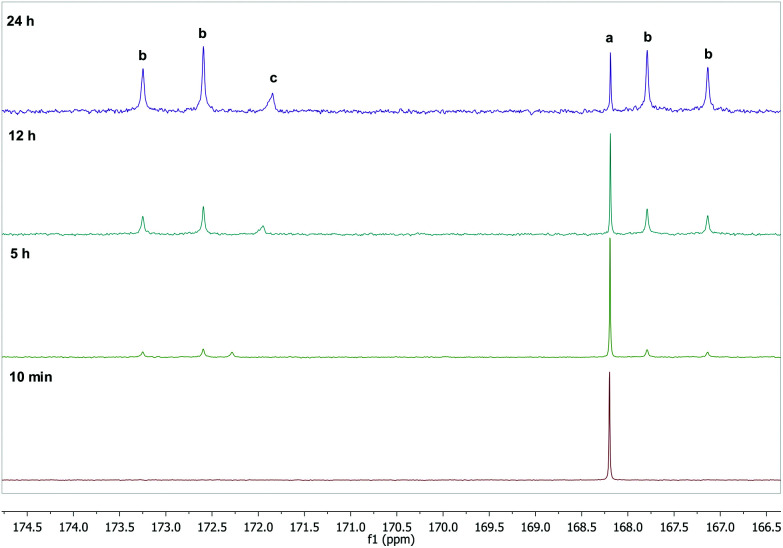
^13^C NMR spectra of oxaliplatin analogue with ^13^C_2_-labeled oxalate in a D_2_O/DMSO-d_6_ (10 : 1) mixture as a function of time at RT (**a**, chelating oxalate ligand; **b**, monodentate oxalate; **c**, uncoordinated oxalate).

The addition of NaCl (5 eq.) led to rapid substitution of the monodentate oxalate and the formation of [Pt(DACH)Cl(DMSO)]^+^, as evidenced from ^13^C and ^195^Pt NMR spectroscopy (Fig. S11†) and ESI MS. Monitoring the reaction of oxaliplatin in DMSO/water mixtures (0.1, 1, 10, 50 and 90% DMSO) shows that the reaction rate increases with increasing concentrations of DMSO, reaching a maximum rate and then decreasing (Fig. 4). The reaction is slow (*t*_1/2_ > 3 days) and incomplete when the amount of water is ≤10%, and is fast (*t*_1/2_ = 3–5 h) when the amount of DMSO is between 10 and 50%.

**Fig. 4:**
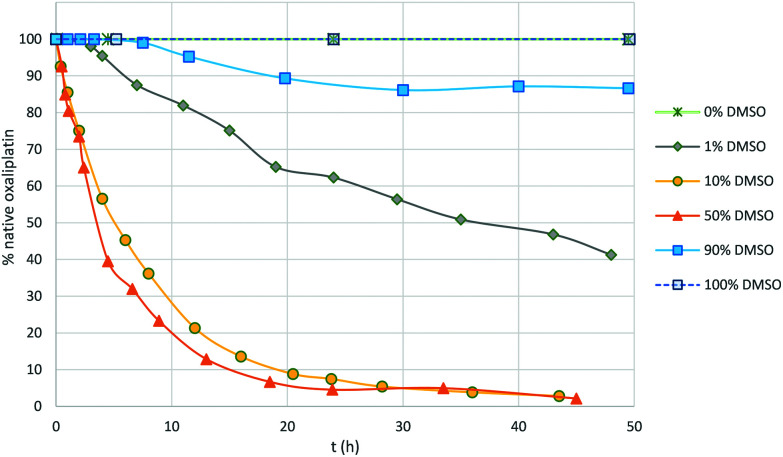
Rate of reaction of the oxaliplatin analogue featuring ^13^C_2_-labeled oxalate and DMSO in different water/DMSO mixtures determined by ^13^C NMR spectroscopy.

The ESI MS and NMR spectroscopic studies described above (see also the scheme in Fig. S12†) show that oxaliplatin does not react with pure DMSO and no evidence was found for Pt-oxalate ring opening. Oxaliplain is also stable in pure water (despite reacting with it) as the reverse ring closure reaction is extremely fast.^23,24^ In DMSO/water mixtures, however, DMSO appears to effectively arrest the ring closure reaction, rapidly coordinating with the ring-opened monoaquated oxaliplatin adduct to form [Pt(DACH)(Ox)(DMSO)]. Notably, only species with one coordinated DMSO were detected, presumably due to the mutual labilization of two *cis*-coordinated DMSO ligands.^25,26^ In DMSO/water solutions [Pt(DACH)(Ox)(DMSO)] is in equilibrium with [Pt(DACH)(OH)(DMSO)]^+^, while the amount of the former species prevails. However, in the presence of chloride [Pt(DACH)Cl(DMSO)]^+^ rapidly forms (Fig S12†).

Carboplatin is also stable in pure DMSO and pure water, but reacts with DMSO in DMSO/water mixtures, nevertheless at a slower rate than oxaliplatin, in accordance with its slower rate of aquation/ring-opening,^23^ due to the higher stability of the six-membered Pt-CBDCA ring, compared to the five-membered Pt-oxalate ring in oxaliplatin. Moreover, the substitution of an ammine ligand by DMSO in carboplatin is favored over the formation of complex with one DMSO and a monodentate CBDCA ligand.

Previous studies reported ring opening of carboplatin, oxaliplatin and related complexes by biologically relevant nucleophiles, *e.g.* 5′-GMP, L-Met, thiourea, GSH, *etc.*, claiming that the major reaction path involves direct nucleophile attack on the intact (non-aquated) platinum complex.^27–29^ However, these studies were performed in aqueous media and, based on the results reported here, the prior formation of monoaquated platinum species cannot be excluded.

In summary, our studies show that carboplatin and oxaliplatin are stable in DMSO in contrast to cisplatin and other dichloridoplatinum(ii) complexes. Nevertheless, DMSO should be avoided in any biological experiments involving carboplatin and oxaliplatin as the aqueous media mediate the formation of DMSO adducts, which are not representative of the clinically used formulations, and is likely to lead to results different from those obtained in the absence of DMSO. In this context, the slightly potentiating effect of DMSO on the cytotoxicity of oxaliplatin observed by Hall *et al.*^9^ may be attributed to the formation of [Pt(DACH)(DMSO)Cl]Cl, a species for which antitumor activity has been reported.^25^

H. V. is indebted to the Austrian Science Fund (FWF) for the financial support (Schrödinger fellowship, Project number: J3577-B13).

## Supplementary Material

DT-046-C7DT01628J-s001
