# Supplementary material for: Oxaliplatin reacts with DMSO only in the presence of water
Source: Dalton Trans. 2017 Jun 27;46(28):8929–32. doi: 10.1039/c7dt01628j (PMC9272996; doi:10.1039/c7dt01628j)
Supplement: DT-046-C7DT01628J-s001 [file DT-046-C7DT01628J-s001.pdf]

Electronic Supplementary Material (ESI) for Dalton Transactions.
This journal is © The Royal Society of Chemistry 2022

Supplementary information

Oxaliplatin reacts with DMSO only in the presence of water

Hristo P. Varbanov*[a,b], Daniel Ortiz[a], Doris Höfer[b], Laure Menin[a], Mathea S. Galanski[b], Bernhard K. Keppler[b], Paul J. Dyson*[a]

[a] Institut des Sciences et Ingénierie Chimiques, Ecole Polytechnique Fédérale de Lausanne (EPFL),

Lausanne, Switzerland. E-mail: paul.dyson@epfl.ch

[b] Institute of Inorganic Chemistry, University of Vienna, Waehringer Strasse 42, A-1090 Vienna, Austria.

E-mail: hristo.varbanov@univie.ac.at

Contents

Page S3	Experimental section
Page S4	Figure S1 with ^{1}H NMR of cisplatin, carboplatin and oxaliplatin in DMSO-d_6
Page S5	Figure S2 with time dependent interactions between cisplatin and DMSO as measured by ESI MS
Page S6	Figure S3 with selected MS peaks related to oxaliplatin-DMSO adducts
Page S7	Figure S4 with time dependent interactions between oxaliplatin and DMSO in aq. media
Page S7	Figure S5 with RP-HPLC-MS chromatogram of oxaliplatin after incubation in H_2O/DMSO for 24 h
Page S8	Figure S6 with RP-HPLC chromatograms of oxaliplatin incubated in H_2O/DMSO and PBS/DMSO mixtures
Page S9	Figure S7 with ^{1}H NMR of carboplatin in D_2O/DMSO-d_6 mixtures
Page S9	Figure S8 with selected MS peaks related to carboplatin-DMSO adducts
Page S10	Figure S9 with RP-HPLC-MS chromatogram of carboplatin after incubation in H_2O/DMSO for 40 h
Page S11	Table S1 with main peaks observed in the ESI MS spectra during the experiments
Page S12	Figure S10 with time course of formation of different species during incubation of oxaliplatin in D_2O/DMSO-d_6 (10:1) as measured by ^{13}C NMR
Page S13	Figure S11 with ^{13}C and ^{195}Pt NMR spectra of oxaliplatin stored in H_2O/DMSO-d_6 after addition of NaCl
Page S14	Figure S12 with scheme summarizing the possible reactions that take place with oxaliplatin in DMSO, water and DMSO/water mixtures.

Experimental section

Cisplatin, carboplatin and oxaliplatin were prepared according to standard literature procedures.[1–3] The synthesis and NMR spectroscopic characterization of [Pt(*trans*-$^{15}N_2$-DACH)($^{13}C_2$)oxalate] is reported in ref [4]. High purity water was obtained from a Milli-Q water system (18.2 MΩ cm, Milli-Q, Merck). All other reagents and solvents were purchased from commercial suppliers and were used as received.

^{1}H NMR spectra were recorded on a Bruker Avance 400 (400 MHz) or a Bruker Avance III 500 (500 MHz) spectrometers; ^{13}C, ^{195}Pt and 2D NMR spectra were recorded with a Bruker Avance III 500 MHz spectrometer at 500.32 (^{1}H), 125.81 (^{13}C) and 107.55 MHz (^{195}Pt). Pt complexes (c ≈10 mM) were dissolved in DMSO-d_6, D_2O, DMSO-d_6/H_2O (1:1) and D_2O/DMSO-d_6 mixtures with DMSO-d_6 content between 1 to 10%, as well as 90%. NMR spectra were recorded at room temperature (25 °C) over a period of 24 h to a few days.

Mass spectrometry was performed on a LTQ Orbitrap FTMS instrument (LTQ Orbitrap Elite FTMS, Thermo Scientific, Bremen, Germany) operated in the positive mode coupled with a robotic chip-based nano-ESI source (TriVersa Nanomate, Advion Biosciences, Ithaca, NY, U.S.A.). A standard data acquisition and instrument control system was utilized (Thermo Scientific), whereas the ion source was controlled by Chipsoft 8.3.1 software (Advion BioScience). Samples were loaded onto a 96-well plate (Eppendorf, Hamburg, Germany) with an injection volume of 5 µL. The experimental conditions for the ionization voltage was +1.4 kV and the gas pressure was set at 0.30 psi. The temperature of ion transfer capillary was 275 °C. The automatic gain control (AGC) target was set to $1x10^{6}$ and the different spectra were obtained in the 80-1000 *m/z* range in the reduce profile mode with a resolution set to 120,000. In all spectra one microscan was acquired with a maximum injection time value of 1000 ms. Data were analyzed using XCalibur (Thermo Scientific) and MS tools available at http://ms.cheminfo.org.

RP-HPLC measurements were performed on an Ultimate 3000 Dionex system controlled by a Chromeleon 6.80 software. Carboplatin and oxaliplatin (c ≈ 1 mM) were incubated in water, water + 1% DMSO, PBS (pH = 7.4) and PBS (pH = 7.4) + 1% DMSO at 25 °C for 24 h. HPLC measurements were performed immediately after dissolving and after 2, 5 and 24 hours applying the following chromatographic conditions: a reversed phase Acquity BEH C18 column (3.0 x 50 mm, 1.7 µm) from Waters as stationary phase, column temperature 25 °C, gradient eluent

condition with MeOH and 0.1% (v/v) FA (0-5 min, 5% MeOH, 5-8 min, 90% MeOH), flow rate 0.4 mL/min, injection volume 2 μL, UV-vis detection at 210, 225, 250 and 300 nm. For some of the experiments, HPLC was coupled also to Advion expressionL CMS mass spectrometer (ESI ion source).

Figures and tables

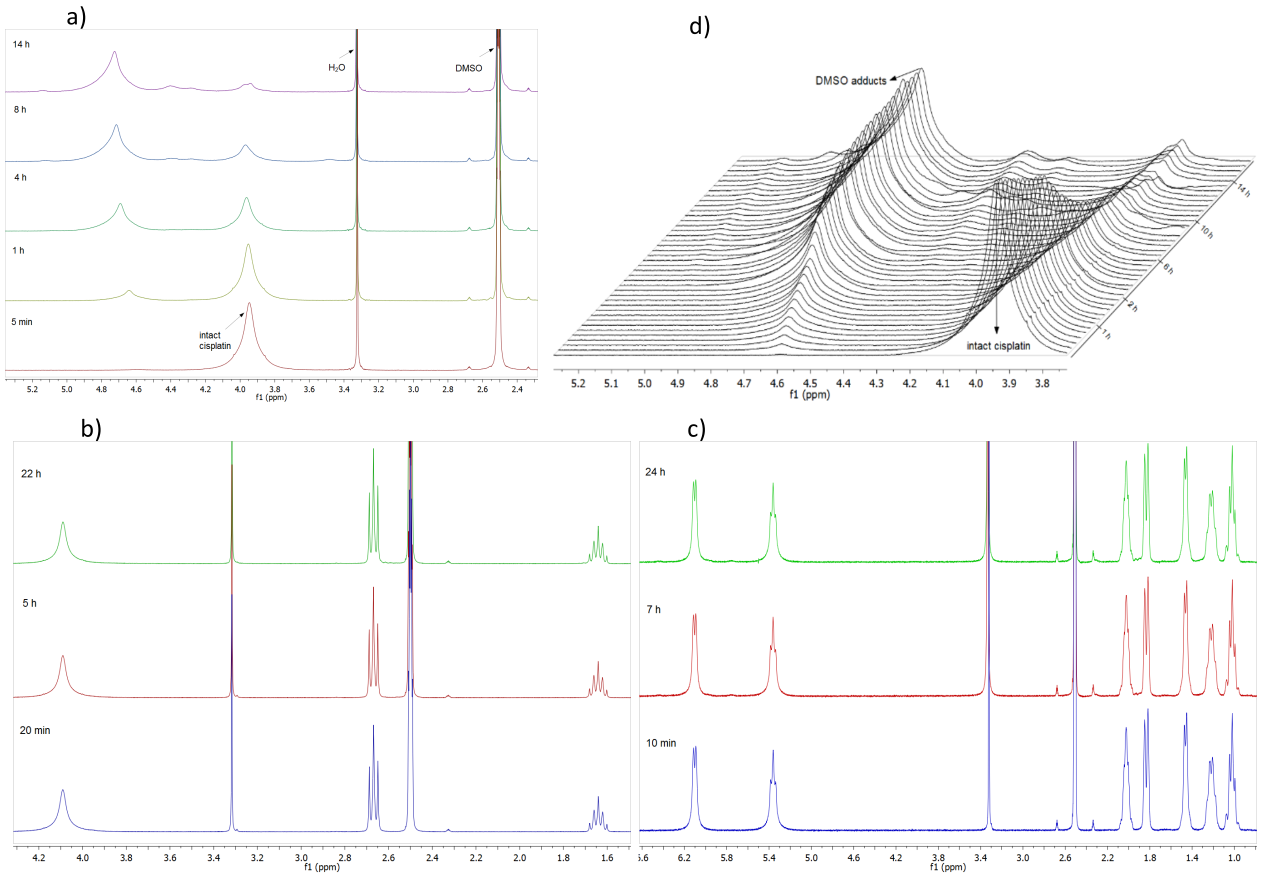

Fig. S1 ^{1}H NMR spectra of cisplatin (a), carboplatin (b) and oxaliplatin (c) in DMSO-d_6 after different periods of incubation at RT. The change of intensity of the NH_3 signal in the ^{1}H NMR spectra of cisplatin in DMSO-d_6, as well as the appearance of new products in solution as a function of time is shown in (d).

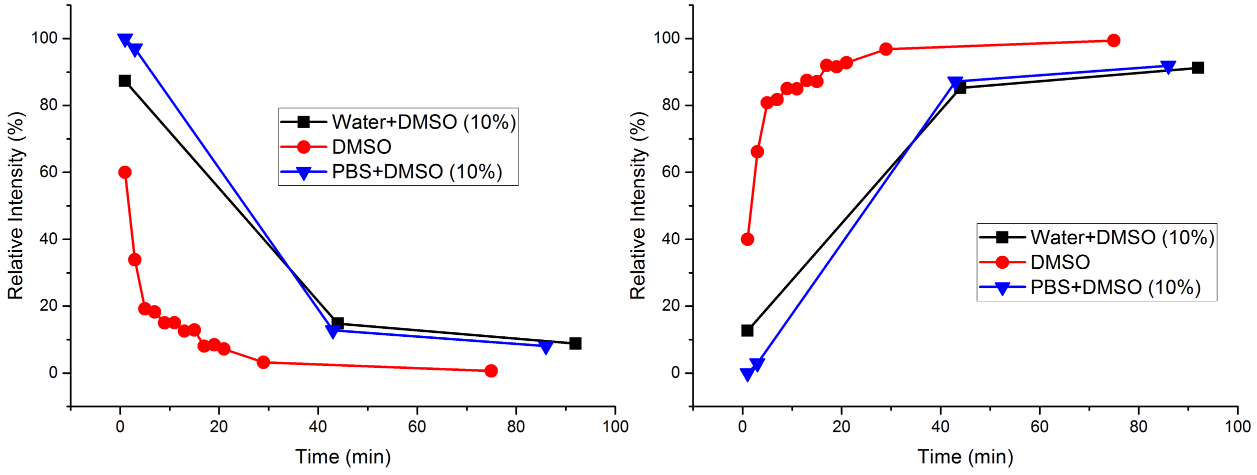

Fig. S2 Rate of consumption of cisplatin (a) and formation of $[Pt(NH_3)_2Cl(DMSO)]^+$ (b) in pure DMSO, clinical formulation (3.3 mM in 0.9 % saline), diluted with water/DMSO (10:1) or with PBS (pH = 7.4)/DMSO (10:1). Aliquots from the respective solutions were taken at different time intervals, further diluted with a MeOH/water (1:10) mixture and measured by ESI MS.

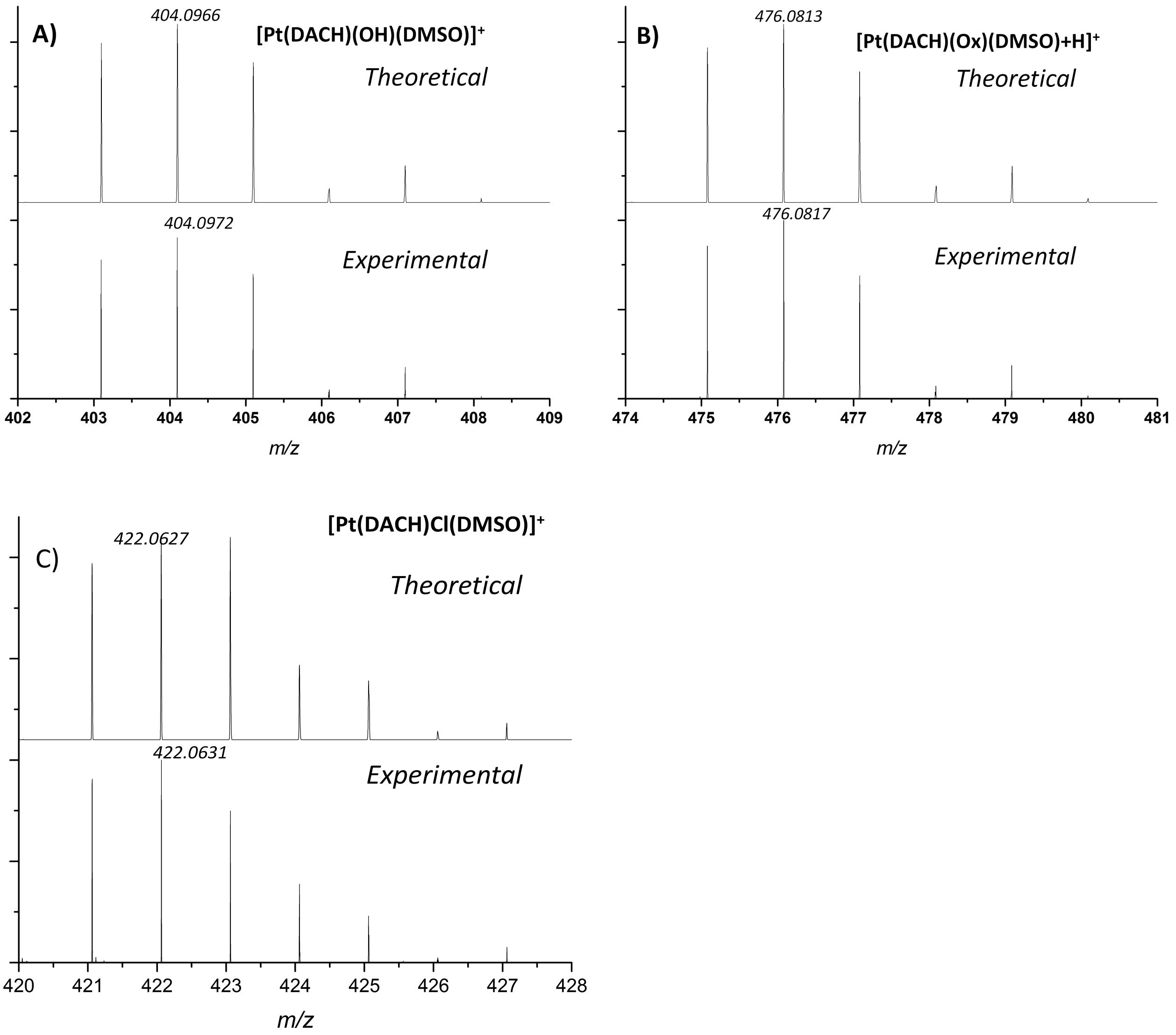

Fig. S3 Selected peaks of oxaliplatin-DMSO adducts observed in the ESI+ mass spectra of oxaliplatin after 10 h of incubation in a water/DMSO (10:1) mixture (A, B) and a PBS (pH = 7.4)/DMSO (100:1) mixture (C); experimental spectrum vs simulated isotopic patterns for $[Pt(DACH)(OH)(DMSO)]^+$ (A), $[Pt(DACH)(Ox)(DMSO)+H]^+$ (B) and $[Pt(DACH)Cl(DMSO)]^+$ (C) are shown.

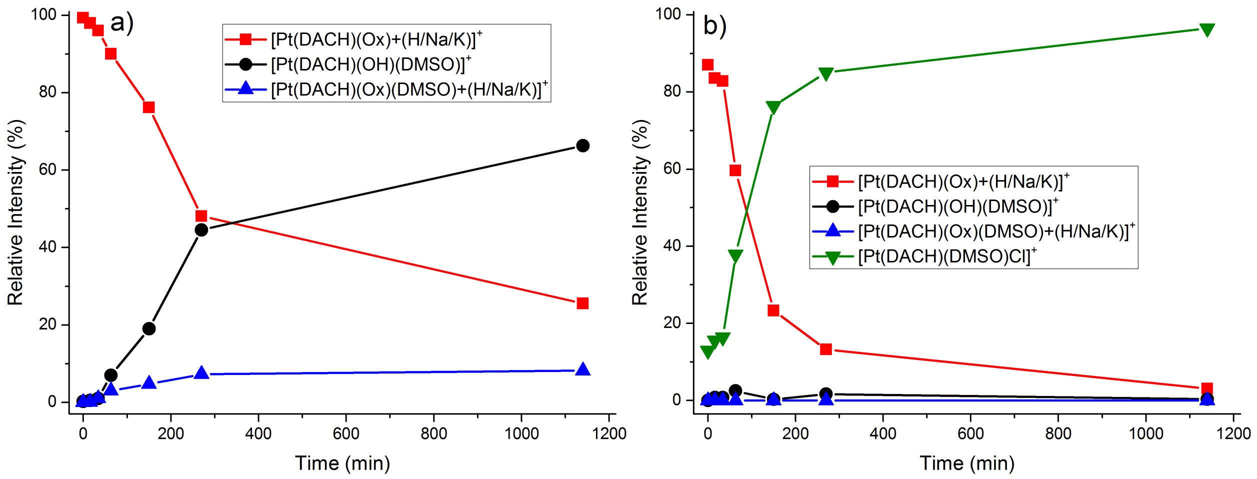

Fig. S4 Time-dependent change in the relative abundance of different species formed in the clinical formulation of oxaliplatin (12.6 mM in 5% glucose solution) after dilution with a) water/DMSO (100:1) or b) PBS (pH = 7.4)/DMSO (100:1) mixtures. Aliquots from the solutions were taken at different time intervals, diluted with MeOH/water and ESI MS were recorded in positive mode.

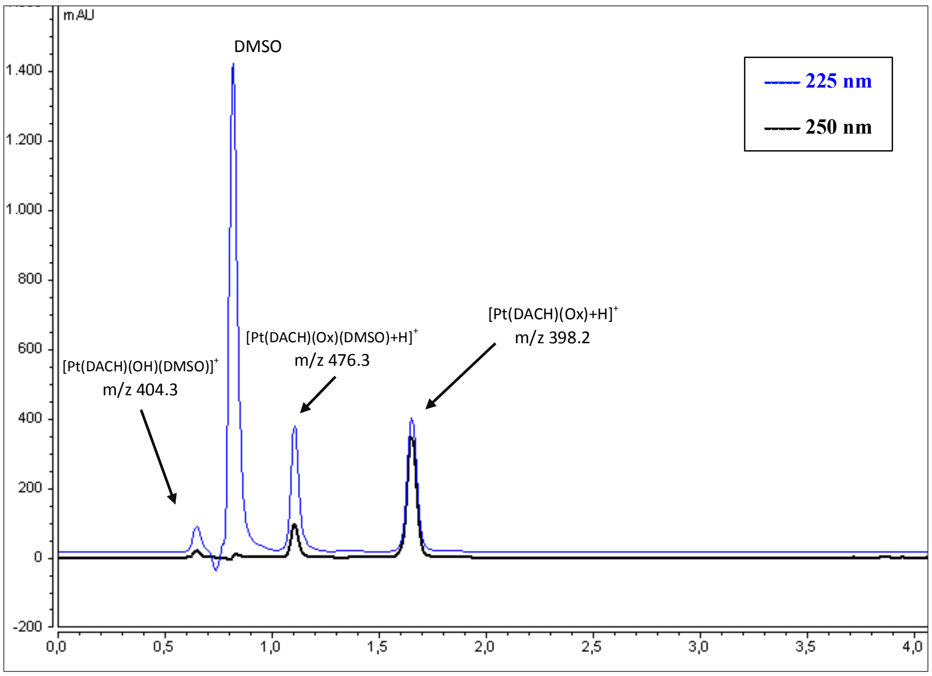

Fig. S5 RP-HPLC-MS chromatogram of oxaliplatin after incubation in water/DMSO (100:1) mixture at 25 °C for 24 h. Overlay of the chromatograms recorded at 225 nm and 250 nm wavelength with the *m/z* values detected for the respective peaks in the MS spectra is shown.

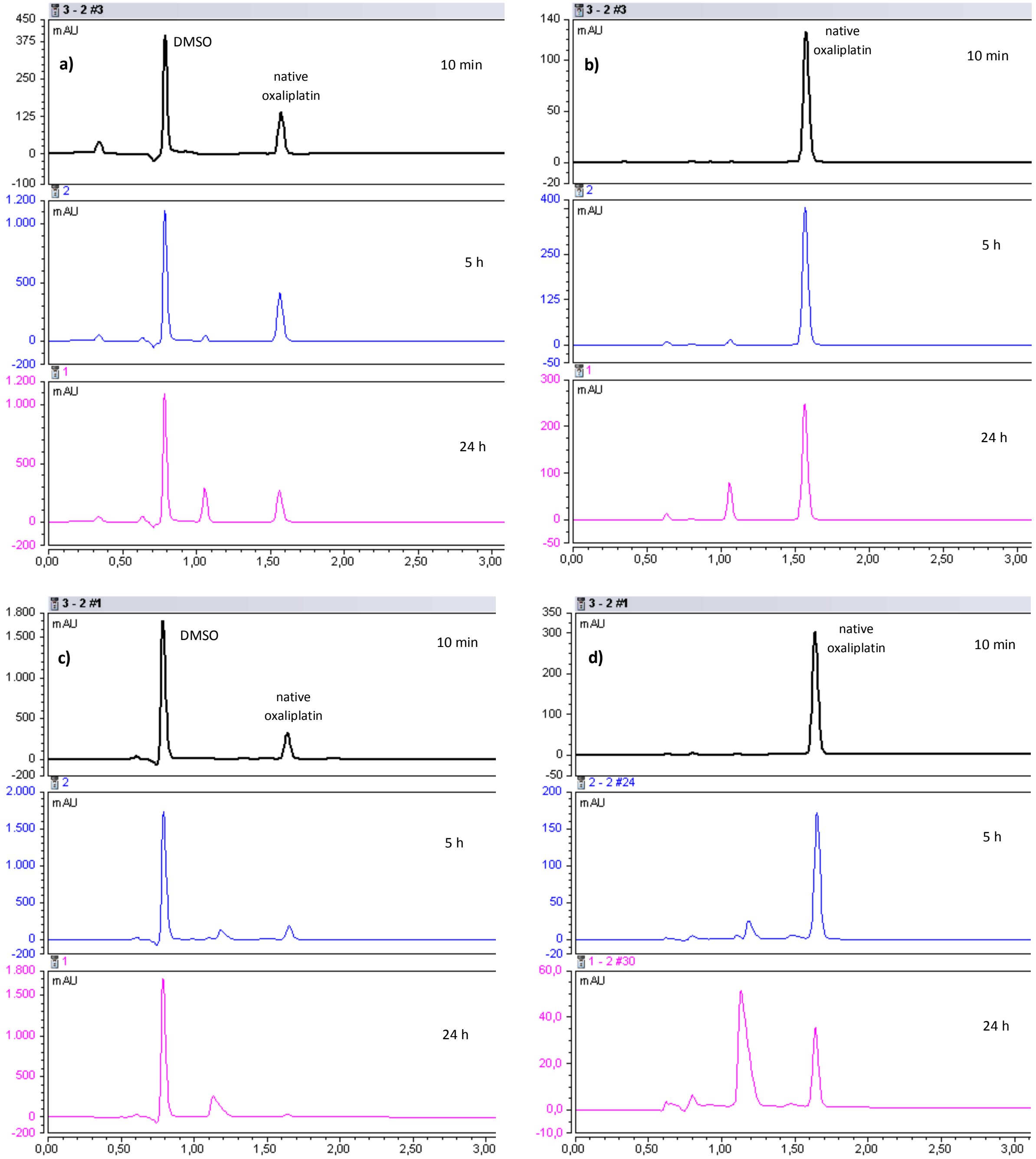

Fig. S6 RP-HPLC chromatograms of oxaliplatin (eluted at 1.6 min) after incubation in water/DMSO (100:1) (a), b)) and PBS/DMSO (100:1) (c), d)) mixtures at 25 °C for 10 min, 5 h and 24 h; UV detection at 225 nm (a, c) and 250 nm (b, d). The decrease of the amount of oxaliplatin after incubation for 24 h was calculated as 46% in water/DMSO and 90% in PBS/DMSO at 225 nm detection wavelength (the respective values were 22% and 75% at 250 nm, respectively). Oxaliplatin is stable in pure water over 24 h whereas < 60% of native oxaliplatin can be found in pure PBS (pH = 7.4) after the same incubation time (data not shown).

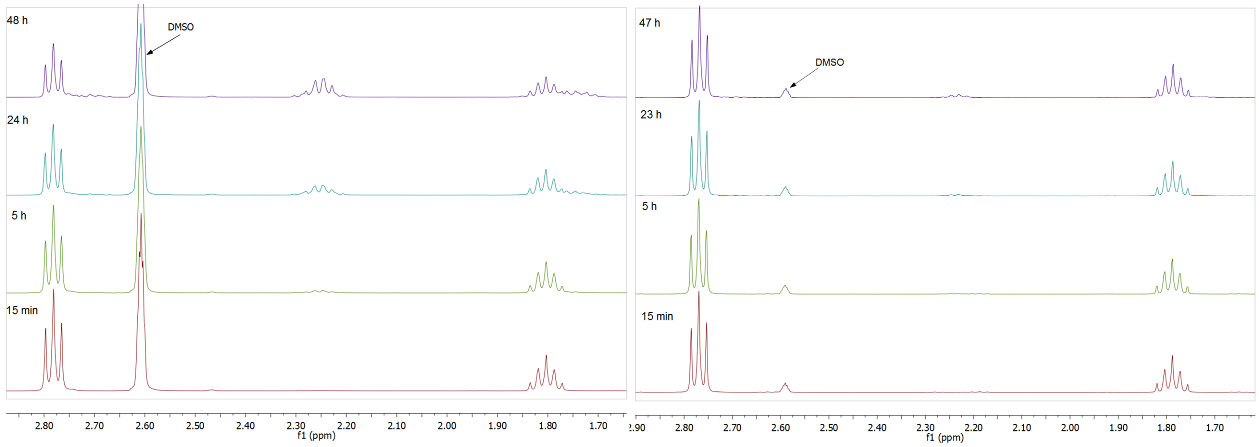

Fig. S7 ^1H NMR spectra of carboplatin in D_2O/DMSO-d_6 mixtures (left, 10:1; right 100:1) after different periods of incubation at RT.

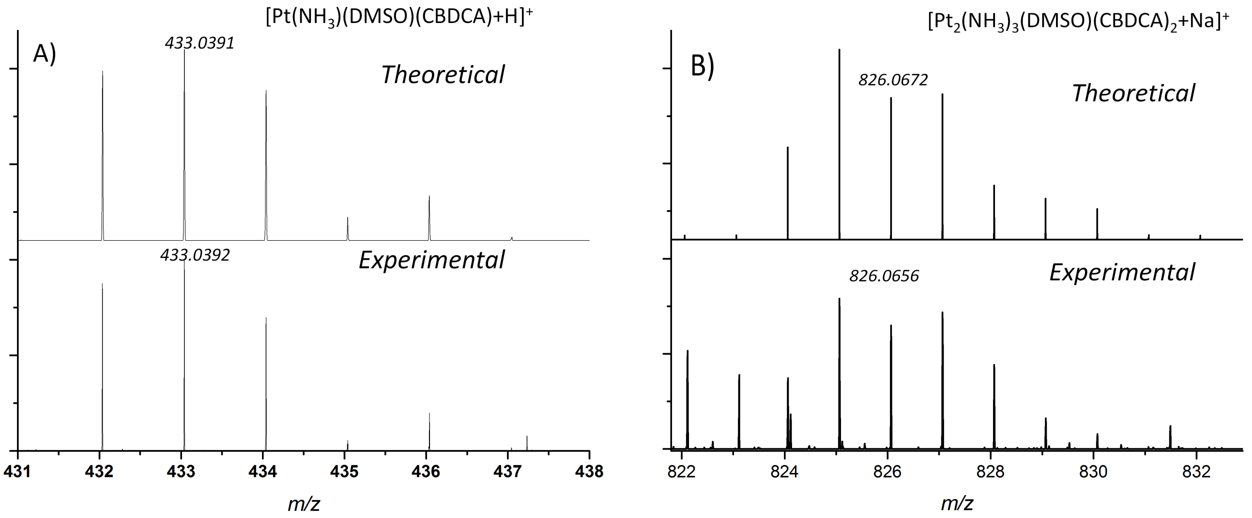

Fig. S8 Selected peaks related to carboplatin-DMSO adducts $[Pt(NH_3)(DMSO)(CBDCA)+H]^+$ (left) and $[Pt_2(NH_3)_3(DMSO)(CBDCA)_2+Na]^+$ (right) observed in the ESI+ mass spectrum of carboplatin after 24 h of incubation in water/DMSO (10:1) mixture; experimental spectrum vs simulated isotopic patterns are shown.

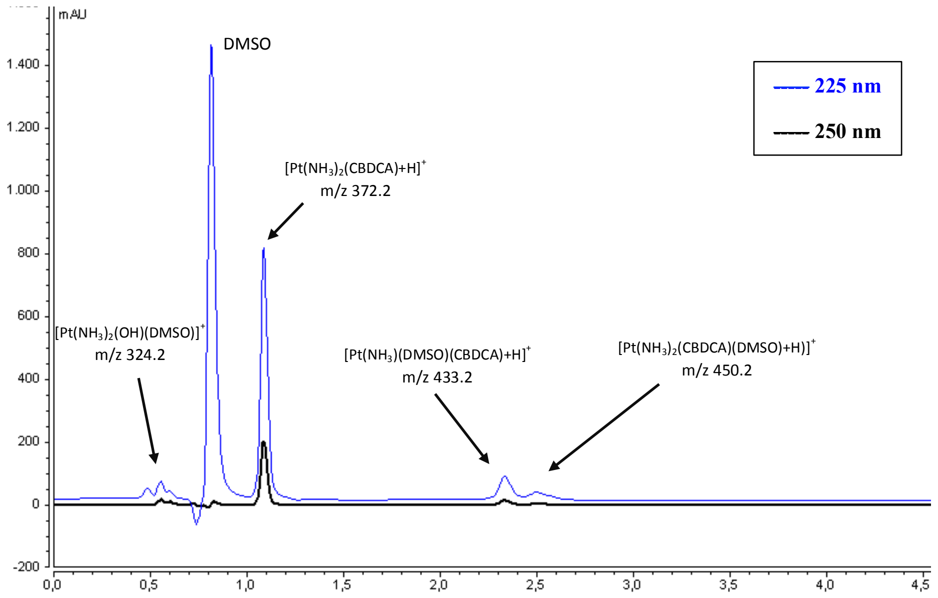

Fig. S9 RP-HPLC-MS chromatogram of carboplatin after incubation in a water/DMSO (100:1) mixture at 25 °C for 40 h. Overlay of the chromatograms recorded at 225 nm and 250 nm wavelength with the *m/z* values detected for the respective peaks in the MS spectra is shown.

Table S1 Main peaks observed in ESI+ mass spectra during the time dependent experiments with the clinical formulations of cisplatin (3.3 mM in 0.9% saline), carboplatin (27 mM in 5% glucose solution) and oxaliplatin (12.6% in 5% glucose solution) diluted with water/DMSO or PBS/DMSO mixtures. Observed/theoretical masses are given for the monoisotopic peaks.

Molecular species	*Formula*	*Observed mass*	*Theoretical mass*	*Error (ppm)*
Cisplatin				
$[Pt(NH_3)_2Cl_2+H]^+$	$H_7Cl_2N_2Pt$	299.9624	299.9629	1.6
$[Pt(NH_3)_2Cl_2+Na]^+$	$H_6Cl_2N_2PtNa$	321.9448	321.9449	0.3
$[Pt(NH_3)_2Cl_2+K]^+$	$H_6Cl_2N_2PtK$	337.9189	337.9187	0.6
$[Pt(NH_3)_2(Cl)(DMSO)]^+$	$C_2H_{12}ClN_2OPtS$	342.0002	342.0001	0.3
$^*[Pt(NH_3)_2(OH)(DMSO)]^+$	$C_2H_{13}N_2O_2PtS$	324.0339	324.0340	0.3
$^*[Pt(NH_3)(Cl)(DMSO)_2]^+$	$C_4H_{15}ClNO_2PtS_2$	402.9873	402.9875	0.5
Carboplatin				
$[Pt(NH_3)_2(CBDCA)+H]^+$	$C_6H_{13}N_2O_4Pt$	372.0520	372.0523	0.8
$[Pt(NH_3)_2(CBDCA)+Na]^+$	$C_6H_{12}N_2O_4PtNa$	394.0339	394.0343	1.0
$[Pt(NH_3)_2(CBDCA)+K]^+$	$C_6H_{12}N_2O_4PtK$	410.0076	410.0082	1.5
$[Pt(NH_3)_2(CBDCA)(DMSO)+H]^+$	$C_8H_{19}N_2O_5PtS$	450.0664	450.0657	1.5
$[Pt(NH_3)_2(CBDCA)(DMSO)+Na]^+$	$C_8H_{18}N_2O_5PtSNa$	472.0483	472.0476	1.5
$[Pt(NH_3)(DMSO)(CBDCA)+H]^+$	$C_8H_{16}NO_5PtS$	433.0393	433.0391	0.5
$[Pt(NH_3)(DMSO)(CBDCA)+Na]^+$	$C_8H_{15}NO_5PtSNa$	455.0218	455.0211	1.1
$^*[Pt(NH_3)(CBDCA)(DMSO)_2+Na]^+$	$C_{10}H_{21}NO_6PtS_2Na$	533.0337	533.0335	0.8
$^*[Pt(NH_3)_2(OH)(DMSO)]^+$	$C_2H_{13}N_2O_2PtS$	324.0339	324.0340	0.3
$[Pt_2(NH_3)_4(CBDCA)_2+H]^+$	$C_{12}H_{25}N_4O_8Pt_2$	743.0973	743.0962	1.4
$[Pt_2(NH_3)_4(CBDCA)_2+Na]^+$	$C_{12}H_{24}N_4O_8Pt_2Na$	765.0794	765.0782	1.5
$[Pt_2(NH_3)_3(DMSO)(CBDCA)_2+H]^+$	$C_{14}H_{28}N_3O_9Pt_2S$	804.0851	804.0836	1.8
$[Pt_2(NH_3)_3(DMSO)(CBDCA)_2+Na]^+$	$C_{14}H_{27}N_3O_9Pt_2SNa$	826.0672	826.0656	1.9
$^*[Pt_2(NH_3)_3(DMSO)(CBDCA)_2+K]^+$	$C_{14}H_{27}N_3O_9Pt_2SK$	842.0413	842.0395	2.1
$^*[Pt_2(NH_3)_2(DMSO)_2(CBDCA)_2+H]^+$	$C_{16}H_{31}N_2O_{10}Pt_2S_2$	865.0735	865.0711	2.7
$^*[Pt_2(NH_3)_4(OH)(DMSO)(CBDCA)]^+$	$C_8H_{25}N_4O_6Pt_2S$	695.0791	695.0785	0.9
Oxaliplatin				
$[Pt(DACH)(Ox)+H]^+$	$C_8H_{15}N_2O_4Pt$	398.0680	398.0678	0.5
$[Pt(DACH)(Ox)+Na]^+$	$C_8H_{14}N_2O_4PtNa$	420.0500	420.0499	0.2
$[Pt(DACH)(Ox)+K]^+$	$C_8H_{14}N_2O_4PtK$	436.0239	436.0238	0.2
$[Pt(DACH)(OH)(DMSO)]^+$	$C_8H_{21}N_2O_2PtS$	404.0972	404.0966	1.4
$^*[Pt(DACH)(OH)(DMSO)-H+Na]^+$	$C_8H_{20}N_2O_2PtSNa$	426.0791	426.0785	1.4
$[Pt(DACH)(Ox)(DMSO)+H]^+$	$C_{10}H_{21}N_2O_5PtS$	476.0817	476.0813	0.9
$[Pt(DACH)(Ox)(DMSO)+Na]^+$	$C_{10}H_{20}N_2O_5PtSNa$	498.0635	498.0633	0.6
$^*[Pt(DACH)(Ox)(DMSO)+K]^+$	$C_{10}H_{20}N_2O_5PtSK$	514.0373	514.0372	0.5
$[Pt(DACH)Cl(DMSO)]^+$	*$C_8H_{20}N_2OPtSCl$*	*422.0631*	*422.0627*	*0.9*

* trace amounts.

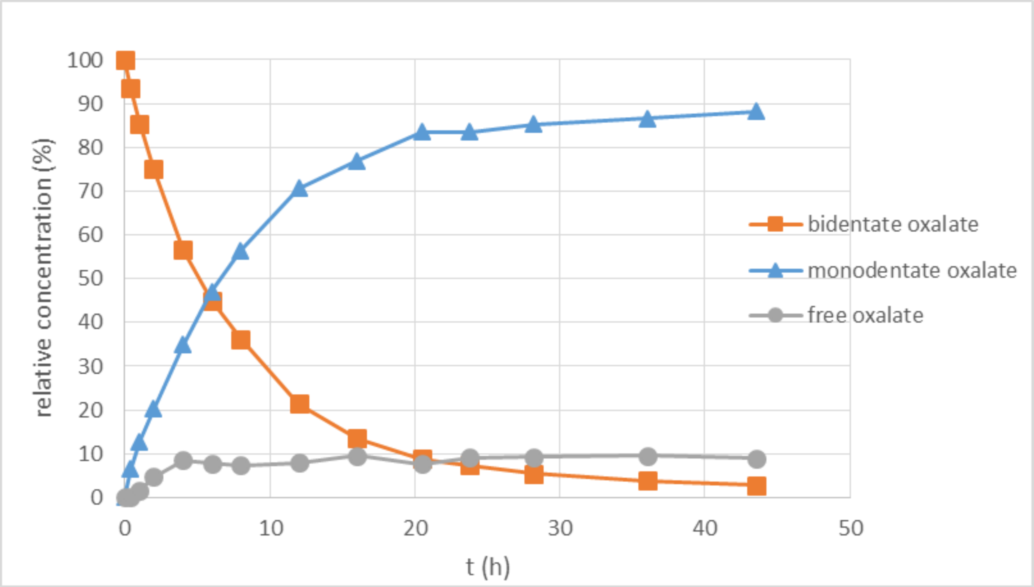

Fig. S10 Time course of formation of different species during the incubation of the oxaliplatin analogue featuring ^{13}C-labeled oxalate in a water/DMSO (10:1) mixture as followed by ^{13}C NMR spectroscopy; bidentately coordinated oxalate in intact oxaliplatin (■), monodentately coordinated oxalate in [Pt(DACH)(Ox)(DMSO)] (▲) and free oxalate (●), corresponding to formation of $[Pt(DACH)(OH)(DMSO)]^{+}$.

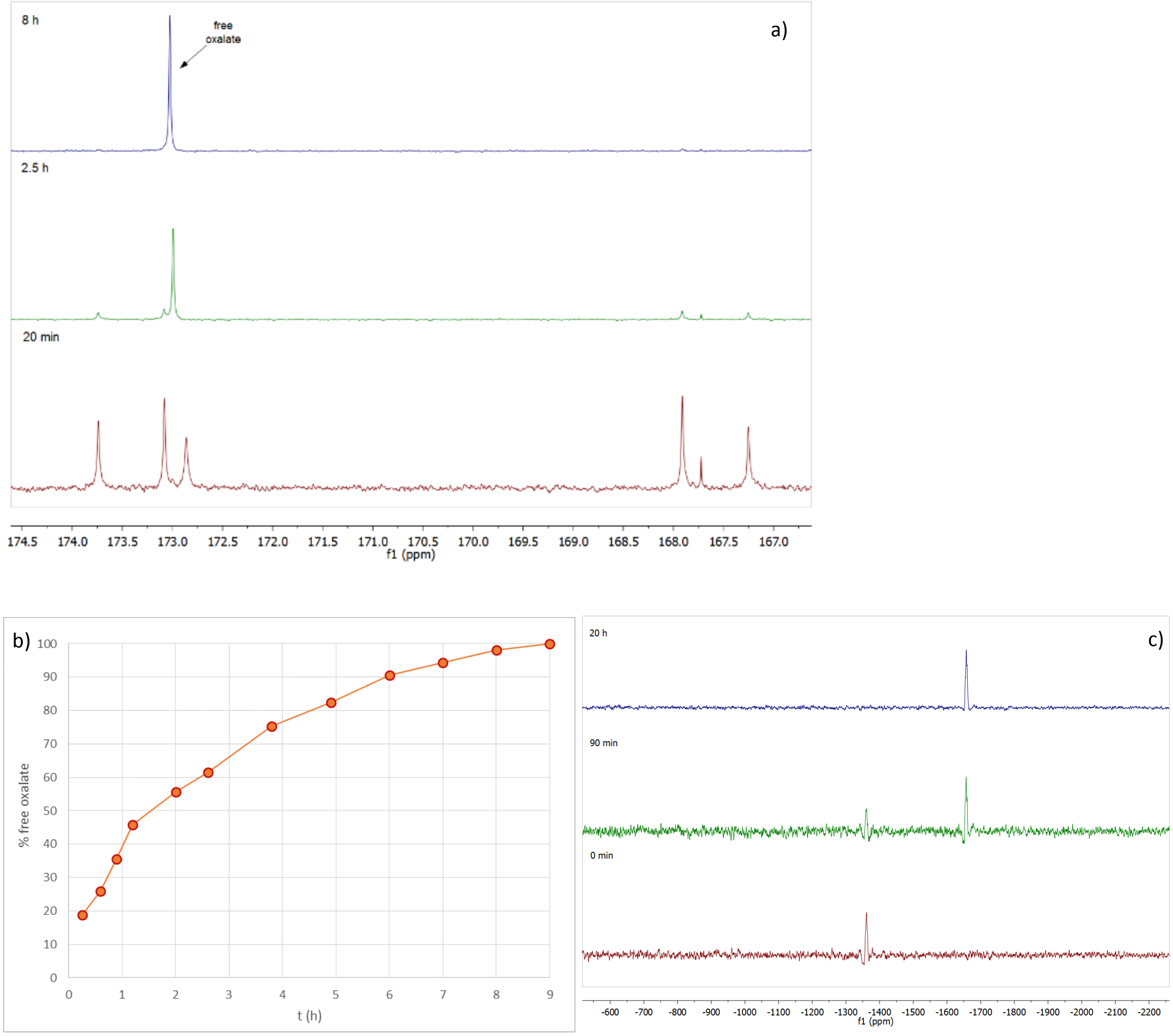

Fig. S11 a) ^{13}C NMR of oxaliplatin analogue with ^{13}C-labeled oxalate stored in H_2O/DMSO-d_6 (1:1) for 4 days at RT after addition of NaCl (5 eq.). **b)** Increase of non-coordinated oxalate formed after the addition of NaCl, determined by ^{13}C NMR spectroscopy. **c)** ^{195}Pt NMR recorded before (point 0), 90 min and 20 h after the addition of NaCl (5 eq.); ^{195}Pt chemical shifts (-1657 ppm and -1361 ppm) were referenced relative to external $K_2[PtCl_4]$.

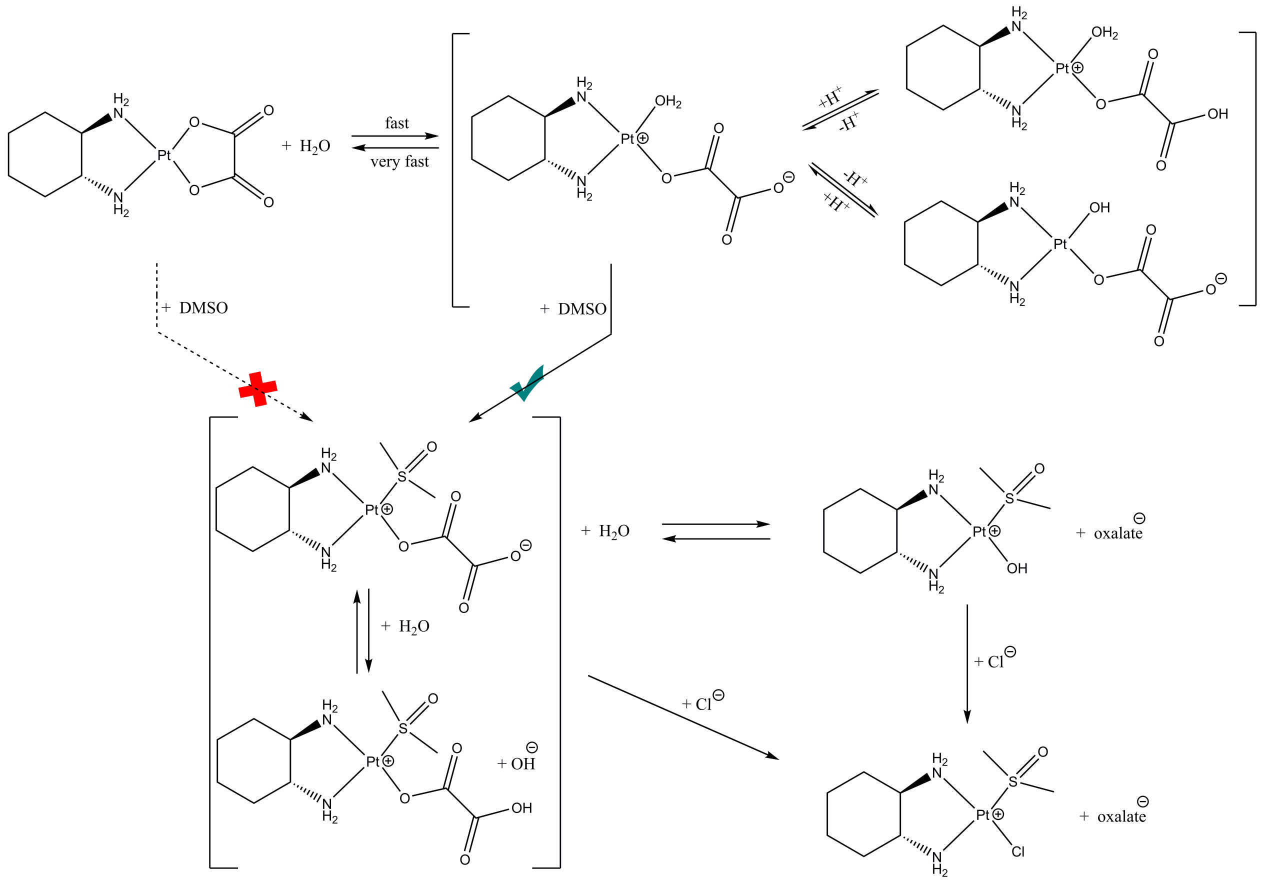

Fig. S12 Scheme summarizing the possible reactions that take place with oxaliplatin in DMSO, water and DMSO/water mixtures. Formation of $[Pt(DACH)Cl(DMSO)]^+$ after addition of NaCl is also noted.

References

(1) S. C. Dhara, *Indian J. Chem.*, **1970**, 193–194.

(2) H. P. Varbanov, S.M. Valiahdi, C.R. Kowol, M.A. Jakupec, M. Galanski, B.K. Keppler, *Dalton Trans.*, 2012, **41**, 14404-14415.

(3) L. Habala, M. Galanski, A. Yasemi, A.A. Nazarov, N.G. von Keyserlingk, B.K. Keppler, *Eur. J. Med. Chem.*, 2005, **40**, 1149–1155.

(4) D. Höfer, M. Galanski, B.K. Keppler, *Eur. J. Inorg. Chem.*, 2017, **17**, 2347-2354.
